# Destabilizing DNA during Rejoining Enhances Fidelity of Repair

**DOI:** 10.1371/journal.pbio.1002214

**Published:** 2015-08-13

**Authors:** Richard Robinson

**Affiliations:** Freelance Science Writer, Sherborn, Massachusetts, United States of America

## Abstract

A new study shows that during repair of DNA, the effect of a single-strand annealing protein is to destabilize DNA duplex formation so that annealing only occurs between perfectly matched strands; the protein then clamps the strands together for repair. Read the Research Article.

Despite its protected position within the nucleus, DNA is subjected to a constant barrage of destabilizing forces, the result of which is an unending series of breaks in the double helix. Repairing the damage is the job of a suite of specialized proteins, which includes a newly discovered superfamily of single-strand annealing proteins (SSAPs). How SSAPs, such as human RAD52 or Redβ from the bacterial phage lambda, function remains unclear, but early studies of RAD52 and Redβ revealed that they could form ring-shaped multimers. Whether and how these ring complexes catalyze DNA repair was puzzling, especially since the high concentrations of protein required to produce the rings also seemed to inhibit, rather than promote, repair.

Now, a new study on Redβ by Marcel Ander, Erik Schäffer, and colleagues shows that the ring appears to play no role in healing broken DNA; instead, repair is initiated by the protein in monomeric form. Surprisingly, the initial interaction between protein and DNA weakens, not strengthens, the link between the two DNA strands, and by the time its job is done, the forces holding Redβ units to one another are among the strongest protein–protein associations known.

The authors measured the force needed to unzip a double-stranded DNA hairpin using “optical tweezers,” whereby one end of the double-stranded DNA was tethered to a moveable surface and the other end to a stationary microsphere, suspended in space by a finely tuned laser, with the hairpin in the middle. Shifting the surface away from the microsphere stretched the DNA, unzipping the hairpin.

The resisting force of the DNA against that stretch was measured over multiple cycles of unzipping and rezipping (annealing) and revealed a surprising pattern. As expected, the presence of Redβ had no effect on the force required for initial unzipping, consistent with its specificity for binding single-, not double-, stranded DNA. Once separated, Redβ bound to the single strands of the separated hairpin. However, when the tweezers relaxed, allowing the hairpin to re-form, the presence of Redβ–impaired annealing, such that the stability of the rezipped structure was less than in the absence of Redβ.

What was the function of this destabilizing effect? Combining measurements and modeling, the authors concluded that the destabilization induced by Redβ promoted fidelity in repair. Without Redβ, even the weak attraction between mismatched DNA bases would bind them tightly together; with Redβ in place, only the best-matched bases stayed bound, ensuring a largely error-free annealing.

Further detailed measurements during annealing showed that multiple Redβ molecules bound at random along the unwound single strands of DNA. Their presence was revealed during unzipping as multiple points of resistance, each about 12 picoNewtons in strength. Over several rounds of zipping and unzipping, the number of bound Redβ molecules increased, until the resisting force jumped from 12 picoNewtons up to 200 picoNewtons, corresponding to the formation of a Redβ dimer. Dimerization secures homology and establishes the nucleus for a nucleoprotein filament (Fig 1). The authors dubbed the very stable complex, caused by a structural change of Redβ, a DNA clamp. This final linkage is among the strongest protein–protein interactions recorded, similar in strength to the attractions between fibrils in insoluble amyloid deposits. As to the ring form of Redβ seen in previous experiments, the authors showed that it only formed at concentrations well above those found in vivo, indicating it was likely an experimental artifact, not a bona fide molecular machine.

**Fig 1:**
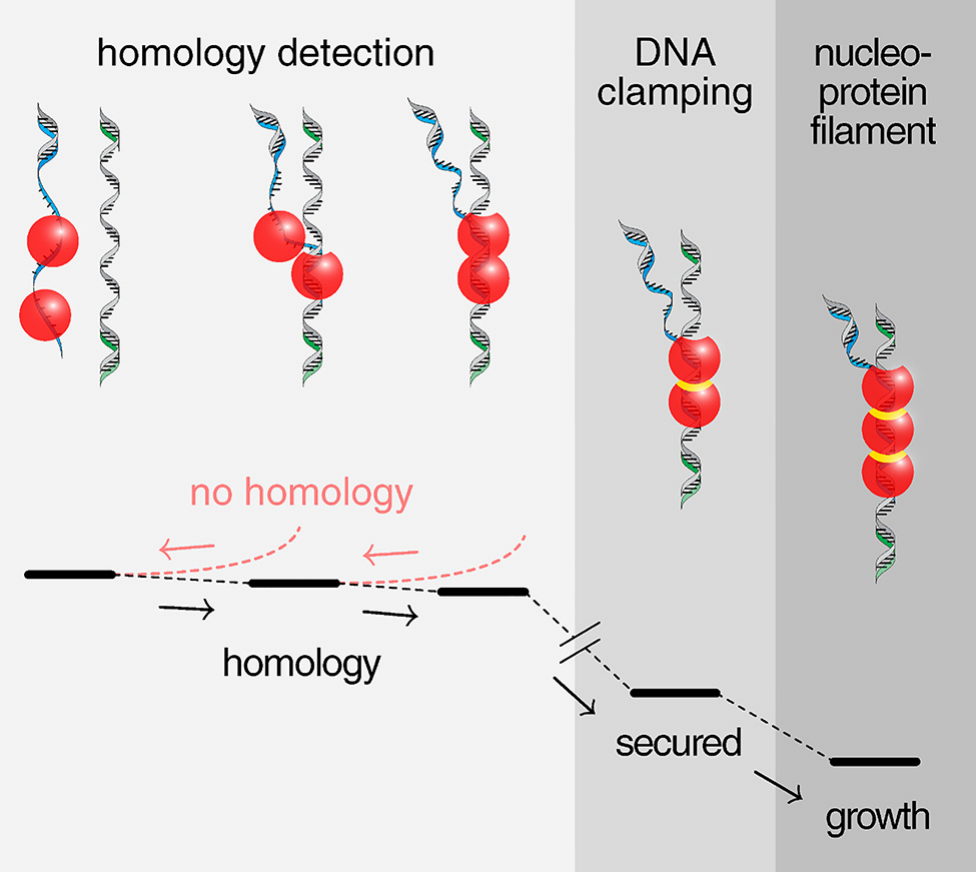
Single-strand annealing. Homology is detected and secured by dimerization of proteins forming an ultrastable DNA clamp. This nucleoprotein complex may resist intracellular pulling forces during mitosis. *Image credit*: *Marcel Ander*.

The strength of the DNA clamp suggests that Redβ or related SSAPs may serve other roles in chromosome dynamics, the authors propose, including maintenance of chromosomal integrity during recombination.

## References

[1] Ander M, Subramaniam S, Fahmy K, Stewart AF, Schäffer E. A single-strand annealing protein clamps DNA to detect and secure homology. PLoS Biol. 2015;13(8): e1002213 doi: 10.1371/journal.pbio.1002213 26271032 10.1371/journal.pbio.1002213 PMC4535883

